# Molecular Detection and Genetic Characteristics of Equine Herpesvirus in Korea

**DOI:** 10.3390/pathogens9020110

**Published:** 2020-02-11

**Authors:** Min-Goo Seo, In-Ohk Ouh, Sang Kyu Lee, Jong-Seok Lee, Oh-Deog Kwon, Dongmi Kwak

**Affiliations:** 1 Animal and Plant Quarantine Agency, 177 Hyeoksin 8-ro, Gimcheon, Gyeongbuk 39660, Korea; koreasmg@korea.kr (M.-G.S.); dvmoio@korea.kr (I.-O.O.); 2 College of Veterinary Medicine, Kyungpook National University, 80 Daehakro, Buk-gu, Daegu 41566, Korea; odkwon@knu.ac.kr; 3 Equine Epidemic Control & Quarantine Section, Korea Racing Authority, Gwacheon, Gyeonggi 13822, Korea; bestvet@kra.co.kr; 4 Horse Riding Military Unit, Korea Military Academy, Nowon-gu, Seoul 01805, Korea; cryboy85@naver.com; 5 Cardiovascular Research Institute, Kyungpook National University, Jung-gu, Daegu 41944, Korea

**Keywords:** herpesvirus, horse, EHV-1, EHV-2, EHV-5, phylogeny

## Abstract

Respiratory diseases cause significant economic losses (especially in the horse racing industry). The present study describes the detection and genetic characteristics of equine herpesvirus (EHV) from a total of 1497 samples from clinically healthy horses in Korea, including 926 blood samples, 187 lung tissues, and 384 nasal swabs. EHV-2 and EHV-5 were detected in 386 (41.7%; 95% CI: 38.5–44.9) and 201 (21.7%; 95% CI: 19.1–24.4) blood samples, respectively, and in 25 (13.4%; 95% CI: 8.5–18.2) and 35 (18.7%; 95% CI: 13.1–24.3) lung tissues, respectively. EHV-1 and EHV-4 were not detected in either blood or lung tissues. EHV-1, EHV-2, and EHV-5 were detected in 46 (12.0%; 95% CI: 8.7–15.2), 21 (5.5%; 95% CI: 3.2–7.7), and 43 (11.2%; 95% CI: 8.0–14.4) nasal swabs, respectively. EHV-4 was not detected in nasal swabs. Co-infection with EHV-2 and EHV-5 was detected in 11.6% (107/926) of the blood samples and 6.4% (12/187) of lung tissues. In nasal swabs, co-infection with EHV-1, EHV-2, and EHV-5 was detected in 0.8% (3/384) of samples. Phylogenetic analysis of the *glycoprotein B* gene showed that EHV-1, EHV-2, and EHV-5 strains demonstrated significant genetic diversity in Korea, with a nucleotide sequence identity among them that ranged from 95.7% to 100% for EHV-1, 96.2–100% for EHV-2, and 93.8–99.3% for EHV-5. These results are the first phylogenetic analyses of EHV-1 in Korea in nasal swabs from a nationwide population of clinically healthy horses. Both EHV-2 and EHV-5 from blood, lung tissues, and nasal swabs were also detected.

## 1. Introduction

In the horse racing industry, infectious respiratory disease caused by viruses is one of the chief factors influencing racing results, causing large economic losses [1]. Equine herpesvirus (EHV)-1 and EHV-4 are Alphaherpesviruses [2] that cause disease in equine populations across the world. EHV-1 infects immune cells; the virus spreads quickly through respiratory mucosal epithelial cells’ basement membrane and causes systemic infection that can result in chronic respiratory disease, abortion, neonatal foal death, chorioretinopathy, and equine herpesvirus myeloencephalopathy [3,4,5]. EHV-4 principally infects epithelial cells and is considered the predominant viral cause of equine acute respiratory disease [6].

Equine Gammaherpesviruses EHV-2 and EHV-5 are associated with enlarged lymph nodes, respiratory pathologies, pharyngitis, fever, lack of appetite, and poor performance in horses [7]. Their true pathogenic importance remains uncertain because of their frequent isolation from healthy horse populations and high seroprevalence globally [8]. However, some reports have linked these EHV strains with respiratory diseases [9,10].

To date, EHV has been detected in Korea: a case of EHV-1 was substantiated by immunohistochemistry and reported in an aborted fetus in 1979 [11]; among horses suffering from respiratory diseases, the presence of EHV-1 and EHV-4 by real-time PCR and EHV-5 by conventional PCR were identified in nasal swab samples in 2008 [12]; and among healthy breeding horses, EHV-2 and EHV-5 were identified in genital swab samples by conventional PCR [13]. The total number of horses in Korea was reported to be 27,829 in 2017, and the horse industry continues to grow annually [14]. Although EHV is important economically, studies on the occurrence and epidemiology of these viruses in Korea remain scarce. The present study sought to discover the epidemiologic parameters of EHV using molecular techniques.

## 2. Results

### 2.1. PCR Detection

The incidences of different EHV strain infections were evaluated from a total of 1497 samples from clinically healthy horses, including 926 blood draws, 187 lung tissues, and 384 nasal swabs. We detected EHV-2 and EHV-5 in blood samples at 41.7% (386; 95% confidence interval: 38.5–44.9) and 21.7% (201; 95% CI: 19.1–24.4), respectively, and in lung tissues at 13.4% (25; 95% CI: 8.5–18.2) and 18.7% (35; 95% CI: 13.1–24.3), respectively (Table 1). EHV-1 and EHV-4 were not detected in either blood or lung tissues. EHV-1, -2, and -5 were detected in nasal swabs at 12.0% (46; 95% CI: 8.7–15.2), 5.5% (21; 95% CI: 3.2–7.7), and 11.2% (43; 95% CI: 8.0–14.4), respectively (Table 2). EHV-4 was not detected in any nasal swabs. Representative photographic images of the results of PCR are described in Supplementary Figure S1.

Unique detection of EHV-2 or EHV-5 in blood samples was identified at 30.1% (279) and 10.2% (94), respectively. Unique detection of EHV-2 and EHV-5 in lung tissues was observed at 7.0% (13) and 12.3% (23), respectively. Co-infections of EHV-2 and EHV-5 were identified at 11.6% (107/926) of the blood samples and 6.4% (12/187) of the lung tissues (Table 3).

Unique detection of EHV-1, -2, or -5 in nasal swabs was observed in 7.3% (28), 0.8% (3), and 3.7% (14), of samples, respectively. Five (1.3%) horses were co-infected with EHV-1 and -2, 16 (4.2%) horses with EHV-1 and -5, and 13 (3.4%) horses with EHV-2 and -5, while three (0.8%) were co-infected with EHV-1, -2, and -5 (Table 3).

The proportion of horses positive for EHV-2 and EHV-5 in blood samples varied for each group (Table 1). Horses under five years old (*p* = 0.0119) were less likely than other age groups to be positive for EHV-5. Central region horses were less likely than horses from other regions to be positive for EHV-2 (*p* = 0.0008) and EHV-5 (*p* = 0.0006). The native Korean ponies (*p* = 0.0008) were more likely than other breeds to be positive for EHV-5.

The proportion of horses positive for EHV-1, EHV-2, and EHV-5 in nasal swabs varied for the different groups (Table 2). Female horses (*p* = 0.0119) were less likely to be positive for EHV-5 than other sex status groups. Horses less than five years of age were less likely than other age groups to be positive for EHV-1 (*p* = 0.007), EHV-2 (*p* = 0.0058), and EHV-5 (*p* < 0.0001). Horses over 10 years old were more likely to be positive for EHV-1 (*p* = 0.0014), EHV-2 (*p* = 0.0008), and EHV-5 (*p* < 0.0001) than other age groups. Northern region horses were less likely than horses from other regions to be positive for EHV-1 (*p* < 0.0001) and EHV-5 (*p* = 0.0092). Southern region horses were more likely to be positive for EHV-2 (*p* < 0.0001) than those from other regions. Racehorses were less likely than other activity groups to be positive for EHV-1 (*p* < 0.0001), EHV-2 (*p* < 0.0001), and EHV-5 (*p* < 0.0001). Breeding horses were more likely than other activity groups to be positive for EHV-1 (*p* = 0.0001) and EHV-5 (*p* = 0.0004).

### 2.2. Molecular and Phylogenetic Analysis

**Figure 1:**
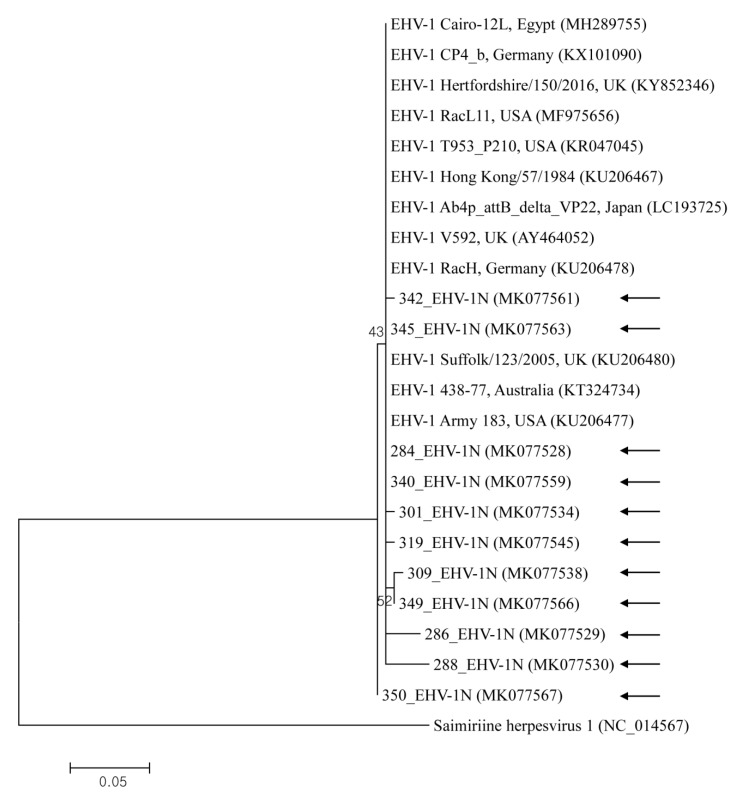
This shows the equine herpesvirus 1 (EHV-1) phylogenetic tree (constructed using the maximum-likelihood method) based on sequences of the *glycoprotein B* gene. Numbers above/beneath branches indicate bootstrap support levels (1000 replicates), and the scale bar shows phylogenetic distance. Black arrows represent the sequences detected in this study, and GenBank accession numbers of other sequences are shown in brackets.

**Figure 2:**
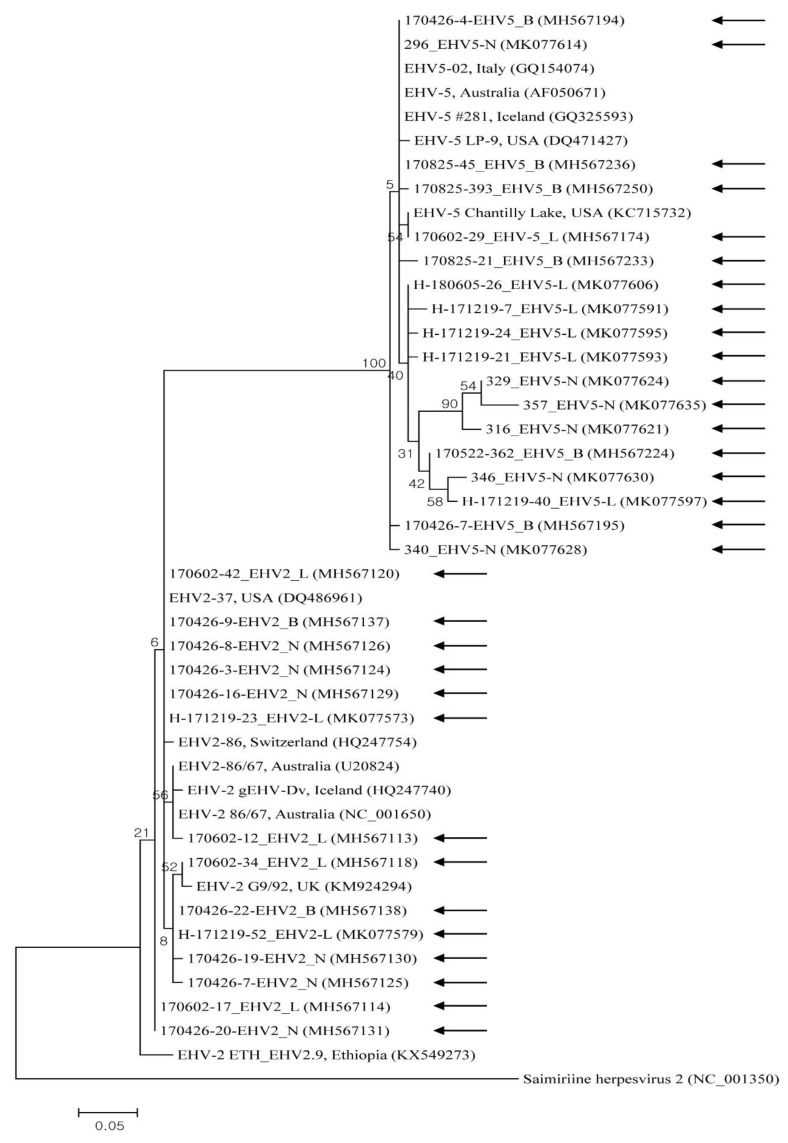
Equine herpesviruses EHV-2 and EHV-5 phylogenic tree (constructed using the maximum-likelihood method) based on *glycoprotein B* gene sequences. Black arrows represent sequences detected in this study, and GenBank accession numbers of other sequences are shown in brackets. The scale bar shows phylogenetic distance, and numbers above/beneath branches show bootstrap support levels (1000 replicates).

EHV-1, -2, and -5 nucleotide sequences reported in the present study were submitted to GenBank under the accession numbers MH567111–MH567253, and MK077526–MK077635. Representative samples selected from the different sample types and rearing regions were used for phylogenetic analysis. The Korean EHV strains demonstrated genetic diversity, with the nucleotide sequence identity among 11 EHV-1 genes, 14 EHV-2 genes, and 18 EHV-5 genes at 95.7–100%, 96.2–100%, and 93.8–99.3%, respectively. Partial nucleotides in the *glycoprotein B* gene of EHV-1, -2, and -5 strains were compared with each other and with previous studies’ sequences of strains (Figure 1 and Figure 2). The gene in EHV-1 shared 97.3–100% identity with those isolated from Egypt, the USA, the UK, Japan, Germany, Australia, and Hong Kong. The gene from EHV-2 shared 91.9–99.8% identity with isolates from Australia, Switzerland, Iceland, and the UK. The gene from EHV-5 shared 95.5–100% identity with genes isolated from Australia, the USA, Iceland, and Italy.

## 3. Discussion

Respiratory pathogens cause serious disease in equine populations worldwide. EHVs are chief viral agents related to equine respiratory problems with varying severity [3,15]. In the present study, EHV-1, -2, and -5 were detected in clinically healthy horses. In accordance with previous observations, Gammaherpesviruses were regularly detected from nasal swabs from horses lacking evident clinical signs [8]. However, at this time, few reports have been published on EHV-2, -5, or other airway diseases of horses [16,17,18].

More recently in other countries, prevalence observed in nasal swab samples from Poland were EHV-2 (77.2%, 417/540), EHV-4 (0.4%, 2/540), and EHV-5 (47%, 254/540) in horses [19], and in nasal swab and blood samples from Ethiopia they were EHV-1 (3.4%, 4/119), EHV-2 (25.2%, 30/119), EHV-4 (7.6%, 9/119), and EHV-5 (28.6%, 34/119) in horses with respiratory disease or EHV-2 (7.9%, 8/101) and EHV-5 (17.8%, 18/101) in healthy horses [20]. In a previous Korean study, EHV-1 (5.6%, 5/89) and -4 (7.9%, 7/89) by real-time PCR and EHV-5 (39%, 35/89) by conventional PCR were detected only from nasal swabs from horses suffering from respiratory diseases in 2008 [12], and EHV-2 (2.3%, 10/430) and -5 (2.6%, 11/430) by conventional PCR only were detected in genital swabs from healthy horses recently [13]. We provide the first phylogenetic analysis of EHV-1 in nasal swab samples from a nationwide (in Korea) population of horses lacking clinical signs of disease, and both EHV-2 and EHV-5 were also first discovered in blood and lung tissue samples.

In this study, EHV-1 only was detected in nasal swab samples, whereas EHV-2 and -5 were detected in blood, lung tissue, and nasal swab samples. The incidence of Gammaherpesviruses was much higher than that of Alphaherpesviruses; this finding recapitulates results from a previous study [8]. According to sample types, the prevalence was the highest in blood, followed by that in lung tissue and nasal swab samples. Results revealed a higher prevalence of EHV-2 (41.7%) than that of EHV-5 (21.7%) in the blood. Previous studies from Hungary, Sweden, the UK [21], New Zealand [16], Poland [19], and Iceland [22] have also reported that EHV-2 is more frequently detected than EHV-5. However, in this study, lung tissue and nasal swab samples presented with a higher prevalence of EHV-5 (18.7% and 11.2%, respectively) than that of EHV-2 (13.4% and 5.5%, respectively); this finding corresponds with reports from Australia [10], Ethiopia [20], Korea [13], and Turkey [18]. When measured in different sample types such as peripheral blood mononuclear cells, conjunctival swabs, nasal swabs, or blood, comparable results were discovered in other countries [8,21,23]. EHV-1 was only detected in nasal swab samples; however, EHV-4 was not detected in any of the samples evaluated in this study. The Alphaherpesviruses are transmitted mostly by nasal secretion and latently infect the trigeminal ganglion [1,6]. It is assumed that the virus can be activated by physical competition or other types of stress, and that virus shedding in nasal secretions varies depending on the individual status of the horse [12].

In the present study, concurrent infections with EHV-2 and -5 were detected in 11.6% of blood samples and 6.4% of lung tissues. Concurrent infections with three different EHV strains were recorded in 0.8% of nasal swabs. Co-infection with EHV-2 and -5 was also found in other studies [18,21,24] reporting that both viruses can concurrently infect horses [20]. Such co-detection of alpha and gamma EHV was also found in nasal swabs in a previous Korean study [12].

Partial sequences of EHV strains’ *glycoprotein B* gene were used to compare their phylogenetic relationships to one another and to reference sequences from GenBank. The phylogenetic analysis of the *glycoprotein B* gene indicated that EHV strains detected in Korea display genetic diversity.

This study describes the first large-scale, nationwide explanation of EHV nucleotides detected in blood, lung tissue, and nasal swab samples from horses without clinical respiratory signs. EHV was geographically distributed across all tested Korean provinces. This investigation suggests that EHV is endemic in Korea and that the effect of viral infections on equine health should be evaluated in more depth. This study offers a basis to further investigate the clinical significance of EHV infection and highlights the need for broad epidemiological studies to clarify the host–pathogen relationship and genetic divergences of EHV to develop effective prevention and control measures. Moreover, further work is required to improve understanding of how EHV establishes pathogenesis.

## 4. Materials and Methods

### 4.1. Ethics Statement

This study was conducted from 2017 to 2018; approval from the Institutional Animal Care and Use Committee (IACUC) at Kyungpook National University (KNU) was not granted, as the KNU IACUC evaluates laboratory animals maintained in indoor facilities (not research involving outdoor animals). Practicing veterinarians at local clinics collected blood and nasal swab samples from clinically healthy horses during regular medical check-ups after receiving verbal consent from farm owners. Clinically healthy horses were slaughtered for horse meat in the local abattoir, and lung tissue samples were collected at that time. 

### 4.2. Sample Size Determination and Sample Collection

The statistical sample size was determined using a formula with a confidence level of 95%, an accepted absolute error of 5%, and an expected disease prevalence of 10% with a simple random sampling design [25].

**Figure 3:**
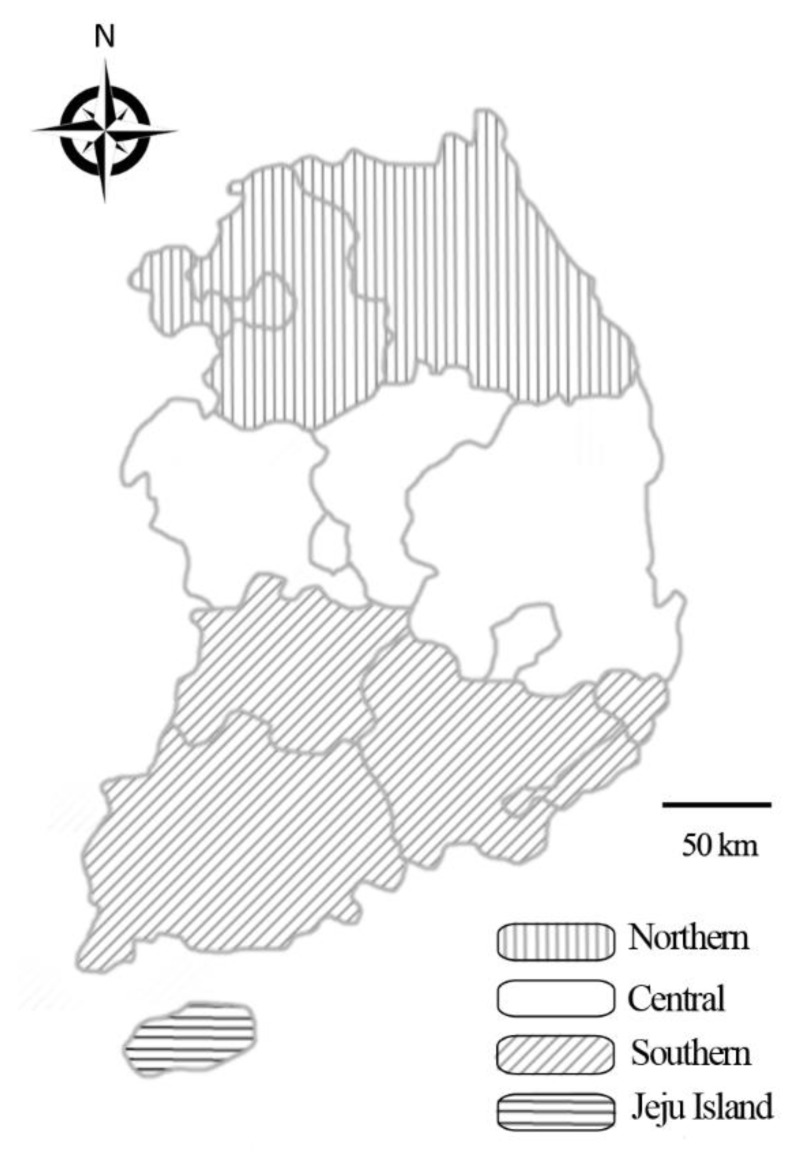
Map of Korea displaying the regions from which horse samples were collected for analysis of the presence of equine herpesvirus: Northern, Central, Southern, and Jeju Island.

According to the formula, a minimum of 138 samples was needed. In present study, we randomly collected 1497 samples (926 whole blood samples from farms, 187 lung tissue samples from the local abattoir, and 384 nasal swab samples from farms) from clinically healthy horses from across the country during 2017 and 2018 (Figure 3). Data on sex, age, breed, activity, and region were recorded for blood and nasal swab samples, whereas the lung tissue samples represented individual horses with no additional data recorded.

### 4.3. DNA Extraction and PCR

Genomic DNA was extracted from samples of whole blood, lung tissues, and nasal swabs using the DNeasy Blood and Tissue Kit (Qiagen, Melbourne, Australia) following the manufacturer’s protocol; quality and quantity were measured with a NanoDrop™ 2000 spectrophotometer (Thermo Fisher Scientific, Wilmington, DE, USA). To identify EHV-1, -2, -4, and -5 in each specimen, we used sensitive one-step PCR targeting the conserved sequence of the *glycoprotein B* gene [20]. PCR amplifications were conducted with a validated pair of primers as described previously, in that individual amplicon size was 190 bp for EHV-1 [26], 444 bp for EHV-2 [10], 677 bp for EHV-4 [26], and 293 bp for EHV-5 [27], for *glycoprotein B* gene detection and nucleotide sequencing. For each PCR reaction, a sample without DNA was used as a negative control. Supplementary Table S1 describes all primers and amplification conditions used for the detection of EHV from horses in the present study.

### 4.4. Cloning

We purified amplified gene fragments using the QIAquick Gel Extraction Kit (Qiagen) and inserted them into a pDrive vector (Promega, Madison, WI, USA) in accordance with the manufacturer’s instructions; we used the resulting constructs to transform *Escherichia coli* DH5α competent cells (Thermo Fisher Scientific). Those were incubated at 37 ℃ overnight, and plasmid DNAs were extracted using a Plasmid Miniprep Kit (Qiagen) in accordance with the manufacturer’s instructions.

### 4.5. Sequencing and Phylogeny

Macrogen (Seoul, Korea) sequenced recombinant plasmids and *glycoprotein B* gene sequences were analyzed via the multiple sequence alignment program CLUSTAL Omega (version 1.2.1). Results of sequence alignments were corrected using BioEdit (version 7.2.5), and phylogeny was performed with MEGA (version 6.0) with the maximum-likelihood method on the basis of the Kimura two-parameter distance model. Aligned sequences were analyzed with a similarity matrix. The stability of the acquired phylogenetic tree was assessed by 1000-replicate bootstrap analysis.

### 4.6. Statistical Analysis

Statistical analysis was done using GraphPad Prism version 5.04 (GraphPad Software Inc., La Jolla, CA, USA). To analyze tables with more than two variables, Pearson’s chi-square test was used; Fisher’s exact test was used to analyze 2 × 2 tables. In order to examine increased risk of infection with EHV within each category, each group was compared to the remaining population enrolled into the study for the pairwise comparisons (*p*-values subject to Bonferroni correction). A relative ratio calculation was also performed. A *p*-value ≤ 0.05 was supposed statistically significant. A 95% CI was also calculated for all estimates.

## Figures and Tables

**Table 1:** Prevalence of equine herpesvirus (EHV) in Korea, as demonstrated by *glycoprotein B* gene detection in horse blood and lung tissue samples, 2017–2018.

Sample	Category	No. Tested	EHV-2				EHV-5			
			No. Positive (%)	RR	95% CI	*p*-Value	No. Positive (%)	RR	95% CI	*p*-Value
Blood	Sex									
	Female	547	234 (42.8)	1.1	0.9–1.2	0.456	129 (23.6)	1.2	1.0–1.6	0.1051
	Male	119	49 (41.2)	1.0	0.8–1.2	0.921	25 (21.0)	1.0	0.7–1.4	0.9056
	Castrated	260	103 (39.6)	0.9	0.8–1.1	0.4585	47 (18.1)	0.8	0.6–1.1	0.1101
	Age (years)									
	<5	223	97 (34.5)	1.1	0.9–1.3	0.5339	35 (15.7)	0.7	0.5–0.9	0.0119 *
	5–10	349	152 (43.6)	1.1	0.9–1.3	0.3723	81 (23.2)	1.1	0.9–1.4	0.4111
	>10	354	137 (38.7)	0.9	0.8–1.0	0.1505	85 (24.0)	1.2	0.9–1.5	0.1899
	Region									
	Northern	248	103 (41.5)	1.0	0.8–1.2	1	54 (21.8)	1.0	0.8–1.3	1
	Central	114	31 (27.2)	0.6	0.5–0.8	0.0008 *	11 (9.6)	0.4	0.2–0.7	0.0006 *
	Southern	168	78 (46.4)	1.1	1.0–1.4	0.1945	37 (22.0)	1.0	0.7–1.4	0.9178
	Jeju Island	396	174 (43.9)	1.1	0.9–1.3	0.2522	99 (22.7)	1.3	1.0–1.7	0.0366
	Breed									
	Thoroughbred	612	256 (41.8)	1.0	0.9–1.2	0.9439	125 (20.4)	0.8	0.7–1.1	0.2066
	Warmblood	61	26 (42.6)	1.0	0.8–1.4	0.8937	13 (21.3)	1.0	0.6–1.6	1
	Native Korean pony	84	41 (48.8)	1.2	0.9–1.5	0.1661	31 (36.9)	1.8	1.3–2.5	0.0008 *
	Mixed	169	63 (37.3)	0.9	0.7–1.1	0.227	32 (18.9)	0.8	0.6–1.2	0.3549
	Activity									
	Race	10	4 (40.0)	1.0	0.4–2.1	1	2 (20.0)	0.9	0.3–3.2	1
	Leisure	613	245 (40.0)	0.9	0.8–1.0	0.1398	122 (19.9)	0.8	0.6–1.0	0.0643
	Breeding	303	137 (45.2)	1.1	1.0–1.3	0.1361	77 (25.4)	1.3	1.0–1.6	0.0617
	Sub-total	926	386 (41.7)		38.5–44.9		201 (21.7)		19.1–24.4	
Lung tissue		187	25 (13.4)		8.5–18.2		35 (18.7)		13.1–24.3	

RR, relative risk; 95% CI, 95% confidence interval. * Results were statistically significant for the following Bonferroni adjusted *p*-values: Sex (<0.0167), Age (<0.0167), Region (<0.0125), Breed (<0.0125), and Activity (<0.0167).

**Table 2:** Prevalence of equine herpesvirus (EHV) in Korea, as demonstrated by *glycoprotein B* gene detection in horse nasal swab samples, 2017–2018.

Category	No. Tested	EHV-1				EHV-2				EHV-5			
		No. Positive (%)	RR	95% CI	*p*-Value	No. Positive (%)	RR	95% CI	*p*-Value	No. Positive (%)	RR	95% CI	*p*-Value
Sex													
Female	130	20 (15.4)	1.5	0.9–2.6	0.1832	10 (7.7)	1.8	0.85–4.1	0.2343	19 (14.6)	1.7	0.9–3.0	0.0821
Male	128	10 (7.8)	0.6	0.3–1.1	0.0949	3 (2.3)	0.3	0.1–1.1	0.0602	5 (3.9)	0.3	0.1–0.7	0.0024 *
Castrated	126	16 (12.7)	1.1	0.6–1.9	0.7411	8 (6.3)	1.3	0.5–3.0	0.6354	17 (13.5)	1.5	0.8–2.6	0.2214
Age (years)													
<5	192	14 (7.3)	0.4	0.2–0.8	0.007 *	4 (2.1)	0.2	0.1–0.7	0.0058 *	7 (3.6)	0.2	0.1–0.4	< 0.0001 *
5–10	127	16 (12.6)	1.1	0.6–1.9	0.8675	7 (5.5)	1.0	0.4–2.5	1	15 (11.8)	1.1	0.6–2.0	0.8636
>10	65	16 (24.6)	2.6	1.5–4.5	0.0014 *	10 (15.4)	4.5	2.0–10.1	0.0008 *	21 (32.3)	4.7	2.7–8.0	< 0.0001 *
Region													
Northern	207	1 (0.5)	0.01	0–0.1	< 0.0001 *	10 (4.8)	0.8	0.3–1.8	0.6542	15 (7.2)	0.5	0.3–0.8	0.0092 *
Central	31	8 (25.8)	2.4	1.2–4.7	0.0212	2 (6.5)	1.2	0.3–4.9	0.6825	5 (16.1)	1.5	0.6–3.5	0.3706
Southern	86	27 (31.4)	4.9	2.9–8.4	< 0.0001 *	6 (7.0)	1.4	0.6–3.5	0.5893	15 (17.4)	1.9	1.0–3.3	0.0507
Jeju Island	60	10 (16.7)	1.5	0.8–2.9	0.2766	3 (5.0)	0.9	0.3–3.0	1	8 (13.3)	1.2	0.6–2.5	0.5122
Activity													
Race	262	15 (5.7)	0.2	0.1–0.4	< 0.0001 *	3 (1.1)	0.1	0.02–0.3	< 0.0001 *	4 (1.5)	0.05	0.02–0.1	< 0.0001 *
Leisure	98	21 (21.4)	2.5	1.4–4.2	0.0018 *	14 (14.3)	5.8	2.4–14.0	< 0.0001 *	30 (30.6)	6.7	3.7–12.4	< 0.0001 *
Breeding	24	10 (41.7)	4.2	2.4–7.3	0.0001 *	4 (16.7)	3.5	1.3–9.7	0.0343	9 (37.5)	4.0	2.2–7.3	0.0004 *
Total	384	46 (12.0)		8.7–15.2		21 (5.5)		3.2–7.7		43 (11.2)		8.0–14.4	

RR, relative risk; 95% CI, 95% confidence interval. * Results were statistically significant for the following Bonferroni adjusted *p*-values: Sex (<0.0167), Age (<0.0167), Region (<0.0125), and Activity (<0.0167).

**Table 3:** Prevalence of equine herpesvirus (EHV) in Korea as indicated by the *glycoprotein B* gene and classified by the number of pathogens detected, 2017–2018.

Sample Type	Classified Infection	Virus	No. Detected (%)
Blood	Total number positive	EHV-2	386 (41.7)
		EHV-5	201 (21.7)
	Unique detection	EHV-2 only	279 (30.1)
		EHV-5 only	94 (10.2)
	Double detection	EHV-2, EHV-5	107 (11.6)
Lung	Total number positive	EHV-2	25 (13.4)
		EHV-5	35 (18.7)
	Unique detection	EHV-2 only	13 (7.0)
		EHV-5 only	23 (12.3)
	Double detection	EHV-2, EHV-5	12 (6.4)
Nasal swab	Total number positive	EHV-1	46 (12.0)
		EHV-2	21 (5.5)
		EHV-5	43 (11.2)
	Unique detection	EHV-1 only	28 (7.3)
		EHV-2 only	3 (0.8)
		EHV-5 only	14 (3.7)
	Double detection	EHV-1, EHV-2	5 (1.3)
		EHV-1, EHV-5	16 (4.2)
		EHV-2, EHV-5	13 (3.4)
	Triple detection	EHV-1, EHV-2, EHV-5	3 (0.8)

## Supplementary Materials

The following are available online at https://www.mdpi.com/2076-0817/9/2/110/s1, Table S1: Primers used for the detection of equine herpesvirus from horses in the present study, Figure S1: Representative photographic images of agarose gel electrophoresis patterns for *glycoprotein B* gene of equine herpesvirus (EHV).
