# Supplementary material for: Molecular Detection and Genetic Characteristics of Equine Herpesvirus in Korea
Source: Pathogens. 2020 Feb 11;9(2):110. doi: 10.3390/pathogens9020110 (PMC7168308; doi:10.3390/pathogens9020110)
Supplement: Supplementary file 1 [file pathogens-09-00110-s001.pdf]

Supplementary Table S1: Primers used for the detection of equine herpesvirus from horses using the glycoprotein B gene.

Virus	Primers	Sequence 5′ to 3′	Amplicon size (bp)	Amplification conditions	Reference
EHV-1	Forward	GCGTTATAGCTATCACGTCC	190	94°C/5 min; 40 cycles: 94°C/30 s, 64°C/30 s, 72°C/90 s; 72°C/10 min	Kirisawa et al., 1993
	Reverse	ATACGATCACATCCAATCCC			
EHV-2	Forward	GCCAGTGTCTGCCAAGTTGATA	444	95°C/5 min; 40 cycles: 95°C/30 s, 60°C/45 s, 72°C/45 s; 72°C/10 min	Diallo et al., 2008
	Reverse	CATGGTCTCGATGTCAAACACG			
EHV-4	Forward	CCTGCATAATGACAGCAGTG	677	94°C/5 min; 40 cycles: 94°C/30 s, 64°C/30 s, 72°C/90 s; 72°C/10 min	Kirisawa et al., 1993
	Reverse	ATACGATCACATCCAATCCC			
EHV-5	Forward	ATGAACCTGACAGATGTGCC	293	95°C/5 min; 40 cycles: 95°C/30 s, 60°C/45 s, 72°C/45 s; 72°C/10 min	Holloway et al., 1999
	Reverse	CACGTTCACTATCACGTCGC			

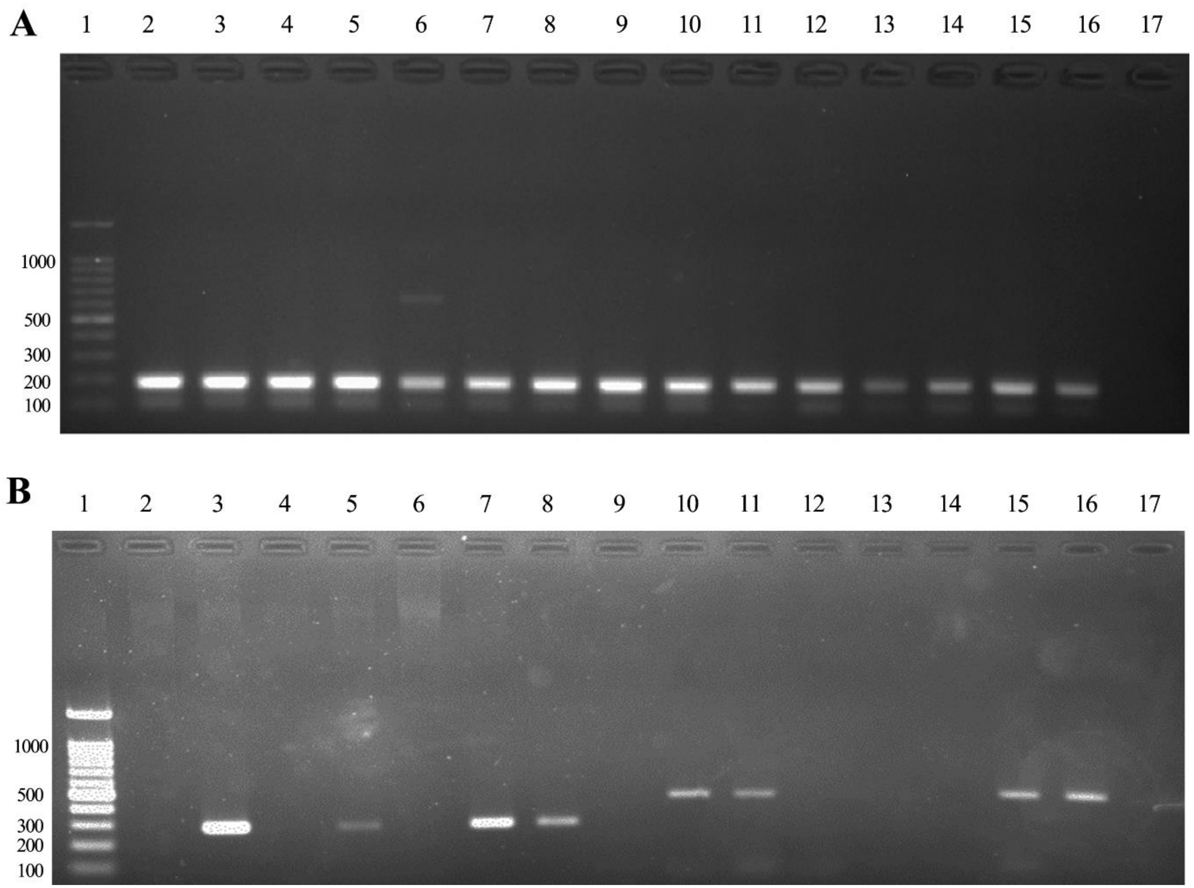

Supplementary Figure S1: Representative photographic images of agarose gel electrophoresis patterns for *glycoprotein B* gene of equine herpesvirus (EHV). (A) PCR products of EHV-1. Lane 1, 100 bp ladder; lanes 2 to 16, EHV-1 PCR products (190 bp) from horse nasal swab samples; and lane 17, negative control. (B) PCR products of EHV-2 and EHV-5. Lane 1, 100 bp ladder; lane 3, EHV-5 PCR products (293 bp) from horse blood sample; lane 5, EHV-5 PCR products (293 bp) from horse lung tissue sample; lanes 7 and 8, EHV-5 PCR product (293 bp) from horse nasal swab samples; lane 10, EHV-2 PCR products (444 bp) from horse blood sample; lane 11, EHV-2 PCR products (444 bp) from horse lung tissue sample; lanes 15 and 16, EHV-2 PCR products (444 bp) from horse nasal swab samples; and lanes 9 and 17, negative controls.
